# Book Review

**Published:** 1932

**Authors:** 


### Reviews of Books

**An Index of Prognosis and End-Results of Treatment.** Editor: A. Rendle Short, M.D., F.R.C.S. Fourth Edition. Pp. xi., 599. Bristol: John Wright and Sons Ltd. 1932. Price 42s.—It is now nine years since the last edition of this book appeared, and not only has every section been thoroughly revised, but so many advances have taken place in the interval that about half the volume is new; for example, the chapter on the outlook in various forms of insanity. The book is more than ever unique and invaluable, for it supplies the reader with a critical review and exact comparison of the chief methods of treatment and their results, not a mere collection of pious opinions. In fact, its aim is, as far as possible, to create an exact science by tabulating all reliable figures as to the duration, mortality and complications of each disease, the precise effects and the percentage of success given by each form of operation or treatment. In short, it would add to Sydenham's "picture of the disease" an engineer's pocket-book of formulæ and statistics. Of course, the possibility of this varies with the disease. Where surgery in recent years has been most successful these statistics are so good and extensive that accurate prognosis can be made easily, but in diseases where science has not yet found a cure or a palliative there is little to be gleaned. Some of the most interesting chapters are those where the claims of medical and surgical treatment are conflicting, such as those on exophthalmic goitre and gastric or duodenal ulcer. Here the wealth of statistics given on both sides is astonishing and the summing up of the end-results is clear and fairly impartial. The value of these sections is very great to every practitioner and not least to the insurance expert. The magnificent Index Series would be sadly incomplete without this crowning volume in its revised form summing up the results of the others.

**The Science of Signs and Symptoms.** By Robert J. S. McDowall, D.Sc., F.R.C.P. Pp. viii., 440. London: William Heinemann Ltd. 1931. Price 21s.—This book is based on the author's *Clinical Physiology* (1927), and is perhaps rather more clinical in scope than the earlier volume.

Its main purpose is to explain on a rational basis the commoner signs and symptoms met with in practice, and to remind the practitioner of relevant physiological facts and principles which may have been crowded out of his memory by other matters, and it should serve its purpose well. But we think it should also prove useful to medical students during their clinical years and to those reading for the higher qualifications in medicine. The book covers a wide field, dealing with a great variety of clinical manifestations and interpreting them in physiological terms, also indicating the physiological basis of treatment in many cases. Perhaps the best sections, as one might expect of the author, are those dealing with the cardiovascular and the respiratory systems; other good chapters are those on gastric ailments, body temperature and fever. As a short appendix to the chapter on feeding, R. D. Lawrence contributes an interesting account of the spontaneous daily food intake of three normal individuals of different types. One would like to see, amongst other things, mention of proteosuria, Kendall's name in connection with the isolation of thyroxine, and fuller treatment of the electrocardiogram, the third nerve nucleus and the Argyll-Robertson pupil. The grouping of subjects is peculiar in some instances; for example, equilibrium and the cerebrospinal fluid are placed together in the same chapter; hypersensitivity, allergy and menstruation in another. On the whole the style is pleasant, making for easy reading. There is a good deal of repetition which may be excused by the attempt to make each chapter independent, though this will not account for exact repetition of whole sentences in the same chapter. There are, unfortunately, a number of misprints, as well as careless mistakes of spelling; the etymologist will hardly approve of "leucopænia," "leucopœnia," "gastrocnæmius," "hæmianopia," and "sella tursica"; and there are many gross spelling errors in the names of chemical substances and in proper names. The printing is good, though the paper employed makes the volume very heavy for its size. The book can certainly be recommended as a very readable link between physiology and practical medicine.

**The Heart and Spleen in Health and Disease.** By G. A. Stephens, M.D. Pp. xi., 139. 6 Figures. London: H. K. Lewis and Co. Ltd. 1932. Price 7s. 6d.—"Hech mon," cried the proud father as the regiment marched by, "they're a' oot o' step but oor Jock!" The author, conscious that his views are unique, anticipates with much reason that

"the Detractor will cut and tear him most cruelly." In fact, one has to search far in this book for a page free from mis-statements positively ludicrous in their naive simplicity. Dr. Stephens maintains that the heart sounds are caused by splashing at the free surface of the pericardial fluid—no free surface, no sounds: the jugular pulse is produced by the heart jerking the wall of the vein; nourishment is conveyed to the tissues by the red corpuscles, and oxygen via the white: "Throughout the whole animal kingdom there is but one virus": disease is an alteration in the rate of the basal exudation (not further defined): cancer is due to lack of oxygen—remedy, drink oxygenated milk: sudden heart failure during anæsthesia should be treated by massage of the spleen! On the subject of intestinal autotoxis, the evils of vinegar, and the dietetic value of uncooked cabbage he is merely tedious: far otherwise when writing of personal and crowd magnetism, telepathy, and the survival of the "aura" after death. There are some startling misprints—or are they such? When one reads that death from one cause is "more chronic" than from another, can one be certain that "the synopses of the autonomic nerves" is not really printed as written? It is seldom indeed that we can approve nothing in a book except the paper and type.

**Sanitary Law in Question and Answer.** 3rd Edition. By Charles Porter, M.D., B.Sc., M.R.C.P., and James Fenton, M.D., D.P.H. Pp. xvi., 219. London: H. K. Lewis & Co. Ltd. 1932. Price 7s. 6d.—This book, now in its third edition, is intended for the medical student and others studying for examinations. The catechism form in which it is written has been found very useful by those who have to acquire a grasp of a difficult subject in a limited time. Sanitary law is found to be such a subject by the average medical student, health visitor, and sanitary inspector. The manual is divided into two parts: Part I deals with Public Health Acts, and Part II with allied acts. This division helps to simplify the subject, which is also covered fairly thoroughly. The book will also be found to be valuable by those who wish to revise the subject.

**Criminal Abortion.** By L. A. Parry, M.D., B.S., F.R.C.S., Pp. viii., 203. London: John Bale, Sons and Danielsson Ltd. 1932. Price 10s. 6d.—Criminal abortion, whether through economic pressure, as well as the stigma attaching to illegitimacy, is considerably on the increase. We therefore welcome this book of Dr. Parry's as filling a gap in the literature on this subject, and as an effort to make practitioners conversant not only with the methods, but with the medico-legal consequences as relating to them. Dr. Parry deals first with the various ways, both by drugs and instruments, for procuring abortion, illustrating them aptly by cases that have been tried in the Criminal Court from time to time. He then summarizes concisely the common and statute law governing this offence, and lastly outlines the steps the practitioner should take when confronted by a case of this sort. We can recommend this very readable volume to all practitioners, and especially to those engaged as police surgeons or as gynecological specialists.

**Individual Sexual Problems.** By F. G. CROOKSHANK, M.D., F.R.C.P. Pp. 150. London: Kegan Paul, Trench, Trubner & Co. Ltd. 1931. Price 2s. 6d.—This small book contains three lectures dealing with sex problems from the standpoint of the Adler school of psychology. The title is perhaps a little misleading to the general reader, owing to the use of the word "Individual," which is used with a particular meaning. Individual psychology implies specifically Adler's school of thought. The book is very well written and interest is sustained throughout. It will well repay the attention of both the medical profession and the intelligent layman. It is full of sound common sense on the upbringing of children, on the conduct of parents, and it contains a brief but very significant résumé of the genesis of neurosis. The fault lies essentially in the way problems of life are tackled rather than in the problems themselves. While Adler admits the importance of the sex factor, for him it is but one of three important factors which contribute towards the total personality. The author summarizes these three cardinal agencies as Society, Sex and Subsistence. The way the individual meets their joint claims is his "Life-style." Perfect adjustment in all the departments makes the well-balanced, healthy individual. Maladjustment, and this includes repression, makes for neurosis. The author brings out well the difference in the Freudian and Adlerian point of view. For Adler it is not the occurrence of a psychical trauma of a sexual kind which is responsible for the development of neurosis, but the way in which the situation is met that matters. How much responsibility for ill-health, unhappiness, and spoilt lives generally rests with parents, doctors, and teachers is clearly brought out. For this reason alone the book deserves to be widely read. The author has succeeded in condensing a maximum of wisdom in a very small space.

**Radium and Cancer.** By H. S. SOUTTAR, C.B.E., M.D., M.CH., F.R.C.S. Pp. viii., 64. London: John Bale, Sons and Danielsson Ltd. 1932. Price 2s. 6d.—The first and the last chapters of this book are well written and easy to understand, but as the author tried to compress a textbook into a pocket monograph, the intervening chapters are neither so well written nor so helpful. It is rather a pity that the author did not confine his remarks solely to the Radon or seed technique, as with the extra space then at his command the book would have been infinitely better. As it is, other methods have been less stressed than seed technique, and the author gives some rather scrappy information. On the whole, this book could only be recommended to those who are already well versed in Radium therapy, and merely wish to brush up their knowledge and get in touch with modern methods.

**Thomson and Miles' Manual of Surgery.** Vols. 2 and 3. By ALEXANDER MILES, M.D., LL.D., F.R.C.S., and D. P. D. WILKIE, M.D., F.R.C.S. Eighth Edition. Pp. xvi., 685; xii., 578. Illustrated. London: Oxford University Press. Price 12s. 6d. each.—The new (eighth) edition of this well-known handbook has been edited by Mr. Miles and Mr. D. P. D. Wilkie, who have enlisted the help of many of the present teachers in the Edinburgh School of Surgery to revise chapters dealing with the regions in which they are especially interested. For many years these handy volumes have been popular with students, and they reflect the sound, careful teaching which has made Edinburgh renowned throughout the surgical world. Mr. Wilkie's own work on obstructive appendicitis and cholecystitis is included, and every chapter shows the imprint of thorough revision. The clinical illustrations and the reproductions of radiographs are extremely clear and well chosen. We can recommend these handbooks to the student and practitioner. To have mastered their contents is to have acquired an up-to-date knowledge of the science of surgery.

**The Technique of the Non-Padded Plaster Cast.** By FRITZ SCHNEK, M.D. Pp. viii., 139. 169 illustrations. Vienna: Wilhelm Maudrich. 1932. Price $5.—During the last few years great interest has been taken in the treatment of fractures by the method of Dr. Boehler of Vienna. Briefly, this consists in reduction by means of powerful skeletal traction and the prevention of any recurrence of deformity by the immediate application of non-padded plaster casts. The fitting of such a splint demands the utmost attention to details of technique, or disaster will follow in the form of gangrene, pressure sores, or non-union. Too little attention has been paid to plaster work in most of our general hospitals in the past, so that a book such as Dr. Schnek's should prove of great value to house surgeons and students. It is a pity that the present work is not more concise. If it were compressed and rewritten in better style it would be much more readily assimilable. It has probably suffered badly in the translation, for it is full of German turns of speech, typographical and grammatical errors. The translator has coined a horrible word "to fixate" which appear over and over again on every page, and the irritation engendered by such mistakes distracts the attention from the valuable matter which the book contains.

**Acute Otitis Media.** Pocket Monograph. By W. M. MOLLISON, C.B.E., M.Ch., F.R.C.S. Pp. vii., 71. Illustrated. London: John Bale, Sons and Danielsson. 1932. Price 2s. 6d.—This little book contains an excellent account of the symptoms, pathology and treatment of acute otitis media and its complications. There is nothing new or startling, but the teaching is throughout clear, concise and modern. We doubt whether the practitioner who needs instruction in the elementary anatomy should be encouraged to perform paracentesis. The author does not allow the diagnostic value to skiagraphy that modern technique deserves, but we are glad to see that he emphasizes the advantages of the magnifying, illuminated otoscope for examination of the membrane. For those who appreciate the "Pocket Monograph," the scope of which is necessarily somewhat restricted, this is a practical and trustworthy guide.

**The Discharging Ear.** Pocket Monograph. By A. G. WELLS, B.S., M.B., J.P., Barrister-at-Law. Pp. x., 92. London: John Bale, Sons and Danielsson Ltd. 1932. Price 2s. 6d.—The title of this book implies a treatise on otorrhœa. Actually, the first quarter is devoted to the external meatus. The author considers the illuminated otoscope "unsuitable" (does he mean "new-fangled"?). The anatomy is described very briefly, not always accurately,

*e.g.*, the nerve supply of the meatus. "Wounds" and "Foreign Bodies" are outside the scope of his title altogether. The second quarter of the book deals with acute otitis media, supplying an unecessary foil to Mr. Mollison's essay on the same subject: skip this section. Chronic suppurative otitis media is described at some length. The *only* method of treatment mentioned is ionization (12 pages), a procedure dreaded by children, employed only occasionally by most otologists, and the darling of the School Authority's heart. The author recommends the risky method of employing "main" current through the patient, and warns the *operator* against receiving shocks: there is one illustration, a rheostat! This book shows the stigma of mass medicine—treatment of "cases" not "patients."

**The Hygiene of Marriage.** By ISABEL E. HUTTON, M.D. Third Edition. Pp. xii., 140. London: William Heinemann Ltd. 1932. Price 5s. This, the third edition of Dr. Hutton's book, fully maintains the good repute of the previous editions. The book is up to date, dealing with the various methods of birth-control as well as the many problems which confront women, and to a certain extent men, before and after marriage. It is written plainly and sensibly, and does not in any way appeal to those in search of erotic literature. The book can be confidently recommended by a doctor to his patients.

**Text-book of Gynæcology.** By SIDNEY FORSDIKE, M.D., F.R.C.S., Pp. xii., 290. Illustrated. London: Wm. Heinemann Ltd. 1932. Price 15s.—This small volume gives but an outline of the subject of gynæcology and can hardly be classed as a text-book. It has, however, some excellent features, the subject-matter is very easily understood and well set out, the diagrams and illustrations are clear and it contains all the recent advances. It would make a good elementary text-book for students and an excellent small reference book for practitioners, to whom it should appeal.

**The Cause of Cancer.** By W. E. GYE, M.D., and W. J. PURDY, M.B. Pp. xiv., 515. London: Cassell and Company Ltd. Price 30s.—The bulk of this book is devoted to experiments on the transmission of avian sarcomata. The authors attack "the entrenched and almost universal views" of the origin of cancer, namely that a change occurs in the nature of the cells, which thereafter multiply uncontrolled. Cancer is a quite sporadic disease with no demonstrable connection between one case and another: so too, they point out, are poliomyelitis and tetanus. Histological evidence of a "cause" or of conversion of normal into malignant cells are lacking, and all attempts to transmit mammalian neoplasms by means of a cell-free extract have failed: merely "negative evidence"! A virus theory supposes a distinct virus for every kind of neoplasm in every species of animal, since each growth "breeds true"; yet they do not blench. They claim to have established that in each avian sarcoma there is (1) a living virus, the essence of neoplasia, non-specific either for type of growth or host; (2) an aggressin, intensely specific, which determines the *type* of growth the virus will produce, and which contains an unaltered molecular group derived from the cell structure. But "as to how the virus enters the body cells when natural infection takes place we have no knowledge." The authors assume that what has been proved for avian sarcoma may be applied without reservation to mammalian tumours, a bridge over which their "old-fashioned" opponents may not readily follow them. While the experimental evidence they adduce can appeal only to those who have the technical knowledge to assess it, the discussion of theories of cancer will interest all readers.

**Research Work on the Pneumococci.** By A. COWAN GUTHRIE, M.B. Pp. ix., 60. Illustrated. London: Baillière, Tindall & Cox. 1932. Price 7s. 6d.—This little book of sixty pages contains the account of a series of investigations, extending over eleven years, undertaken by a medical practitioner in active practice. The experiments were devised to determine the reason why an immune serum is lethal to an homologous organism "in vivo" when the same micro-organism can grow and multiply on the immune serum "in vitro." The work is set forth in detail, and the results are considered in relation to the therapy of lobar pneumonia. The chief features are the isolation of pneumococcal enzymes and their bio-chemical reactions. Stress is laid upon the action of "hæmatoxin" on the blood. The author is to be congratulated upon his perseverance and ingenuity. The book, however, needs to be read critically. It has, perhaps, been presented in its present form with such an end in view.

**A Guide to Anatomy.**—Third Edition. By E. D. Ewart. Pp. xii., 338. Illustrated. London: H. K. Lewis & Co. Ltd. 1932. Price 12s. 6d.—This book makes no claim to being in any sense a systematic text-book for medical students, but is especially written for students of Medical Gymnastics, Massage and Medical Electricity. It contains an enormous amount of information, accurately put together and simply arranged. It contains so much that one cannot resist a feeling of sympathy with the embryo masseuse, who may well feel appalled to find what she is in for. Will she be any better a masseuse for having learned (and forgotten) the surface-marking of the pulmonary valve? However, if it is in the syllabus it must be learned, for it may be asked: so it must go into a book of this kind. The book is an excellent one for the purpose indicated, and the illustrations, though simple, are satisfactory and adequate: the same applies to the index. The exiguous and inaccurate paragraph on page 175, referring to the surface-marking of the pleura, should be corrected and amplified, or omitted altogether. On the whole, a book well suited to its purpose, which will no doubt be welcomed by those for whom it is written.

